# Hear Less, Feel Less: One Mutation Causes Loss of Two Senses

**DOI:** 10.1371/journal.pbio.1001322

**Published:** 2012-05-01

**Authors:** Richard Robinson

**Affiliations:** Freelance Science Writer, Sherborn, Massachusetts, United States of America

## Abstract

Hearing and touch are genetically related, and people with excellent hearing are more likely to have a fine sense of touch and vice versa.

[Fig pbio-1001322-g001]Take two pins and touch them to the tip of your finger. If they are far apart, you'll have no trouble feeling their two distinct pricks on your skin. But move them closer, and eventually that distinction is lost, and you feel only one point pressing into your finger. That threshold of tactile acuity averages around 1.6 millimeters, but it differs from person to person. Why? In this issue of *PLoS Biology*, Henning Frenzel, Gary Lewin, and colleagues show the answer is at least in part genetic. They report that mutations in at least one gene correlate with reduced touch sensitivity, and, most intriguing of all, that the gene is also responsible for a form of hereditary deafness. That's probably no coincidence, they argue: hearing, like touch, depends on cells exquisitely sensitive to changes in pressure. Thus, Frenzel et al. appear to have found a gene underlying the general ability to transduce physical stimulation into sensory perception.

**Figure pbio-1001322-g001:**
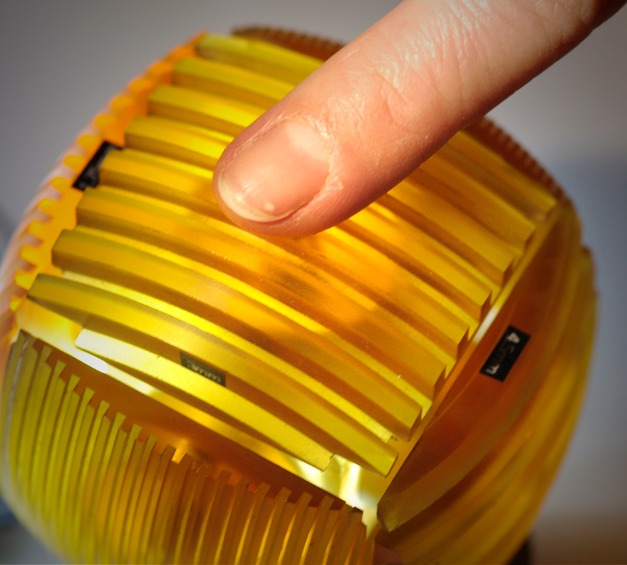
Measuring touch performance with a tactile acuity cube. A person is asked to judge whether the grid is horizontal (pictured) or vertical, the narrower the grid the more difficult the task.

There are a large number of genes for congenital deafness, but, perhaps surprisingly, none known for touch insensitivity. The authors began their search for such genes by first showing they were likely to exist. They performed two tests of touch sensitivity—a test of tactile acuity using a grid of points, similar to the pinprick test, and a vibration threshold test—on pairs of twins, both monozygotic (MZ) and dizygotic (DZ). Since MZ twins share all their genes, while DZ twins share, on average, only half, traits governed by genes should be more strongly similar between MZ twins than between DZ twins.

They found that on both tests, the correlation in performance between MZ twins was twice as strong as between DZ twins, indicating strong genetic effects. They calculated that the heritability, or the fraction of the trait dictated by genes, was 0.28 for tactile acuity, and 0.52 for vibration detection. They concluded that genes for touch sensitivity indeed existed. They found even stronger heritability of various measures of hearing; not surprising, given the large number of genes known to cause deafness. Next, through various statistical tests, they showed that good hearing tended to go along with good touch sensitivity, suggesting the existence of genes influencing both traits.

But twin studies and statistical correlations cannot identify those genes, so the authors next examined touch sensitivity in a population of individuals with Usher syndrome, characterized by early onset deafness. Nine different genes are known to cause Usher syndrome, all of them affecting the pressure-sensitive stereocilia of the inner ear. The authors found that in individuals carrying two mutated copies of the *USH2A* gene, touch sensitivity was diminished compared to controls, with a tactile acuity threshold of about 1.91 mm versus 1.63 mm for controls, and a vibration sensitivity threshold also sharply increased.

Because of the brain's remarkable ability to remold its connections through experience, the loss of one sense can lead to increased sensitivity in another; indeed, the authors found that in a group of blind individuals, the mean tactile acuity threshold was significantly lower than for controls (i.e., their acuity was greater), and some people with Usher syndrome due to other causes performed no worse or even slightly better than controls on these tests. But when a single gene underlies the function of two senses, as appears to be the case with *USH2A*, mutation diminishes them both. There are likely to be other such genes awaiting discovery.

Exactly how *USH2A* mutation affects either sense is unknown. The encoded protein, usherin, is found at the base of the stereocilia, where it may serve to link other cellular proteins to the extracellular matrix, a function perhaps well-suited to transferring extracellular mechanical distortion into the cell, where it could help influence membrane depolarization. Whatever the actual mechanism, the finding that it plays a role in touch will likely trigger important research into its precise function, especially since skin is a more accessible target for experiment than the inner ear.

**Frenzel H, Bohlender J, Pinsker K, Wohlleben B, Tank J, et al. (2012) A Genetic Basis for Mechanosensory Traits in Humans. doi:10.1371/journal.pbio.1001318.**

